# Erythropoietin Derived Peptide Improved Endoplasmic Reticulum Stress and Ischemia-Reperfusion Related Cellular and Renal Injury

**DOI:** 10.3389/fmed.2020.00005

**Published:** 2020-01-24

**Authors:** Yufang Zhang, Qian Wang, Aifen Liu, Yuanyuan Wu, Feng Liu, Hui Wang, Tongyu Zhu, Yaping Fan, Bin Yang

**Affiliations:** ^1^Renal Group, Basic Medical Research Centre, Medical College of Nantong University, Nantong, China; ^2^Department of Nephrology, Nantong-Leicester Joint Institute of Kidney Science, Affiliated Hospital of Nantong University, Nantong, China; ^3^Department of Cardiovascular Sciences, University of Leicester, University Hospitals of Leicester, Leicester, United Kingdom; ^4^Department of Urology, Zhongshan Hospital, Fudan University, Shanghai, China; ^5^Shanghai Key Laboratory of Organ Transplantation, Shanghai, China

**Keywords:** apoptosis, CHOP, cyclic helix-B surface peptide (CHBP), endoplasmic reticulum stress (ERS), inflammation, ischemia-reperfusion injury

## Abstract

Ischemia-reperfusion (IR) injury often affects transplant and native kidneys alike. IR injury is one of the main causes of acute kidney injury (AKI) and further associated with the progression of chronic kidney disease. Our previous study revealed the renoprotection of erythropoietin derived cyclic helix-B surface peptide (CHBP) against IR injury. However, the precise role and underlying mechanism of endoplasmic reticulum stress (ERS) in the injury and the renoprotection induced by IR or CHBP, respectively, have not been fully defined. This study using mouse kidney epithelial cells (TCMK-1) revealed that the level of CHOP (a key marker of ERS), PERK, and JNK (regulators of CHOP) was gradually increased by the prolonged time of hydrogen peroxide (H_2_O_2_) stimulation. In addition, CHOP mRNA and protein were significantly reduced by small interfering RNA (siRNA) target CHOP, as were apoptotic and inflammatory mediator caspase-3 and HMGB-1, and early apoptosis. Furthermore, *CHOP* mRNA was correlated positively with PERK protein, active caspase-3, HMGB-1 and apoptosis, but negatively with cell viability *in vitro*, while CHOP protein was also correlated positively with the level of tubulointerstitial damage and active caspase-3 protein *in vivo*. Finally, CHBP improved the viability of TCMK-1 cells subjected to H_2_O_2_ stimulation time-dependently, with reduced level of *CHOP* mRNA. CHBP also inhibited the increase of CHOP protein, not only in TCMK-1 cells, but also in the IR injury kidneys at 2 weeks. Moreover, CHBP reduced the expression of *PERK* mRNA and protein, JNK and HMGB-1 protein, as well as early and later apoptosis. In addition, raised CHOP at 12 h post IR injury might be an early time window for intervention. In conclusion, the differential role of ERS and CHBP in IR-related injury was proved in mouse TCMK-1 cells and kidneys, in which the mechanistic signaling pathway was associated with CHOP/PERK/JNK, HMGB-1/caspase-3, and apoptosis. CHOP might be a potential biomarker and CHBP might be therapeutic drug for IR-induced AKI.

## Introduction

Renal ischemia-reperfusion (IR) injury is inevitable in kidney transplantation, which greatly affects the survival of allograft (1–3). IR injury also often occurs in native kidneys subjected to a variety of causes such as cardiovascular surgery, dehydration, and sepsis. IR injury is a major inducement of acute kidney injury (AKI) and further associated with its progression to chronic kidney disease (CKD). It is imperative to disclose the mechanism of IR injury in order to achieve time diagnosis and specific treatment for IR-induced AKI and prevent CKD. Renal tubular epithelial cells (TECs) are most vulnerable to renal IR insult, underwent to autophagy, apoptosis or necrosis, which contributes to the loss of renal function, and subsequent repair/recovery or fibrosis (4–6). The underlying mechanisms of IR injury and repair are complicated including endoplasmic reticulum stress (ERS), mitochondria dysfunction, cell death, and inflammation (7–10). Improving ERS and TEC apoptosis have been considered to be effective methods to alleviate renal IR injury (3, 11).

Newly synthesized proteins fold in the lumen of ER prior to exit into various compartments of the cell and extracellular space. In response to adverse conditions, these proteins could folded incorrectly and unable to exit, but accumulate inside ER resulting in ERS (9, 12). A series of adaptions were subsequently activated, called the unfolded protein response (UPR) (9, 13, 14). The UPR is attributed to the activation of one ER transmembrane protein, named protein kinase RNA-like ER kinase (PERK) (15–17). Growing evidence has shown that ERS associated with apoptosis and inflammation plays important roles in IR injury (18, 19). UPR could gradually develop into apoptosis and further induces inflammation, if ERS is too severe to overcome. CCAAT/enhancer-binding protein homologous protein (CHOP) is an ERS-induced transcriptional regulator and key to ERS-mediated apoptotic pathway (20, 21). The over expression of CHOP plays an important role in the activation of C-Jun N-terminal kinase (JNK) that contributes to apoptotic cell death (22, 23). CHOP-mediated apoptosis and inflammation exacerbate myocardial IR injury, whereas CHOP^−/−^ mice have been shown to be resistant to apoptosis in various disease models (24).

Erythropoietin (EPO), on the other hand, could protect various organs including kidney against IR injury (25). The renoprotection of EPO is through a heterodimer EPO receptor and β-common receptor (EPOR/βcR). EPOR/βcR is pharmacologically distinct from the homodimer (EPOR)_2_ that mediates erythropoiesis (26–28). The renoprotection of EPO against IR injury has been shown by our previous studies, in terms of reducing tubular cell apoptosis, but promoting inflammatory apoptotic cell clearance (29, 30). However, the large dosage of EPO was required to achieve tissue protection, which often causes hypertension and thrombosis *in vivo* (31). Therefore, a helix B surface peptide (HBSP) derived from EPO was developed, which is composed by 11 amino acids (QEQLERALNSS) and interacts with EPOR/βcR only, but with very short serum half-life (32). A metabolic stable cyclic HBSP (CHBP) was further generated, having prolonged half-life and potent tissue protection. The renoprotection of HBSP and CHBP against IR injury has also been confirmed in our previous research projects (33, 34). The pretreatment of CHBP reduced the active level of oxidative damage and ERS induced hydrogen peroxide (H_2_O_2_) in human proximal tubular cell line, HK-2 cells (35), but its underlying mechanisms have still not been well-defined, especially the impact of CHBP on ERS regulators in TECs, as well as in kidneys post IR injury with extended time to 2 and 8 weeks.

We hypothesized that ERS and CHBP plays differential role in renal IR-related injury, which is associated CHOP and its regulators, apoptotic and inflammatory mediators, as well as subsequent apoptosis. To verify this hypothesis, TCMK-1 cells, mouse kidney epithelial cell line, stimulated by H_2_O_2_ and mouse kidneys subjected to IR injury were used to evaluate the change of CHOP, the key marker of ERS, and its regulators PERK and JNK, and their relations to apoptotic and inflammatory mediators caspase-3 and HMGB-1, and apoptosis. The impacts of intervention using siRNA target CHOP and CHBP treatment, on above biological events were further studied. It has been proved in this study that ERS plays key roles in IR-related injury and CHBP induced renoprotection in mouse TCMK-1 cells at the early stage and in kidneys with prolonged IR time, and the underlying mechanistic pathway is associated with CHOP/PERK/JNK, HMGB-1/ caspase-3, and apoptosis. CHOP might be a potential biomarker and CHBP might be therapeutic drug for IR-induced AKI.

## Methods and Materials

### TCMK-1 Cell Culture and H_2_O_2_ Stimulation

TCMK-1 cells (a mouse kidney epithelial cell line, CCL-139™, American Type Culture Collection, Manassas, USA) were cultured in DMEM/F12 medium, supplemented with 10% (v/v) of fetal bovine serum (FBS, Gibco Technologies, Logan, USA), 100 unit/ml penicillin G (Gibco), and 100 μg/ml streptomycin (Gibco), defined as completed medium, at 37°C in 5% CO_2_ atmosphere. TCMK-1 cells grown about 80% confluent in monolayer in 6-well plates were treated with or without 500 μmol/L H_2_O_2_ (Merck KGaA, Darmstadt, Germany) diluted in basic culture medium. At 4, 8, 12 h after the exposure of H_2_O_2_, the cells were washed three times with PBS (Ji Nuo, Hangzhou, China) and harvested for further analysis. Twenty ng/ml of CHBP (synthesized by Shanghai Institute of Materia Medica, Chinese Academy of Sciences) was added at the same time upon H_2_O_2_ stimulation and cultured for 12 h (35).

According to the instruction of manufacturer, the small interfering RNA (siRNA) was diluted to 5 nM transfected into TCMK-1 cells using Lipofectamine^@^ RNAiMAX (Invitrogen, Carlsbad, USA). A double-stranded CHOP siRNA (Thermo Fisher Scientific, Waltham, USA) targeting mouse *CHOP* mRNA was designed and constructed. The sequences of CHOP siRNA are: sense: GGAAGAACUAGGAAACGGATT, antisense: UCCGUUUCCUAGUUCUUCCTT. The negative control siRNA (# 4390843, Thermo Fisher Scientific) does not target any known mammalian genes. The transfected cells were cultured at 37°C for 8 h before 12-h H_2_O_2_ stimulation.

### Renal IR Injury Model

Male BALB/c mice weighing 20–25 g were obtained from the Experimental Animal Center of Nantong University, China. They were housed in constant temperature (25°C) and humidity (55%) on a 12-h light/dark cycle, fed *ad libitum* on standard laboratory mouse chow and tap water. All animal procedures were performed according to the guidelines of the Animal Care and Use Committee of Nantong University and the Jiangsu Province Animal Care Ethics Committee (36).

The mice were anesthetized using 80 mg/kg sodium pentobarbital by intraperitoneal injections. The bilateral renal pedicles were exposed by flank incision and clamped for 30 min. For reperfusion, the clamping was released and confirmed blood reflow by monitoring the color change of the kidney before suturing the incision. The mice were randomly divided into 7 groups with different reperfusion time: control, 6, 12, 24, 72 h, and 1 w or 3 groups (*n* = 6): (1) Control group: the abdominal cavity and renal pedicles were exposed without clamping-induced IR injury, (2) IR group: mice were subjected to ischemia 30 min followed by reperfusion for 2 and 8 weeks, (3) IR + CHBP group: IR injury with 24 nmol/kg CHBP dissolved in 0.9% saline intraperitoneally injected after reperfusion every 3 days (37).

### Cell Viability Analysis

TCMK-1 cells (1 × 10^5^) were seeded in 96-well plates and cultured in the completed medium 24 h before adding 500 μmol/L of H_2_O_2_ apart from the control group. In the treatment groups, 20 ng/ml of CHBP was added at the same time upon H_2_O_2_ stimulation. Ten microliter of CCK-8 (Beyotime, Nantong, China) was then added after 1, 4, and 12 h and further incubated for 1–4 h. The cell viability was measured by absorbance using a microplate reader (MDC, Hayward, USA).

### Quantitative Real-Time Polymerase Chain Reaction

Total RNA was extracted from cultured cells with Trizol reagent (Invitrogen) according to the manufacturer's instructions. Complementary DNA (cDNA) was synthesized using 1 μg of total RNA, oligo d(T) 18 Primer and Reverse Transcription Kit (Bio-Rad, Hercules, USA). Quantitative RT-PCR analysis was performed in the real time PCR system (CFX96, Bio-Rad) with AceQ® qPCR SYBR® Green system (Vazyme biotech, Nanjing, China). The oligonucleotide primers for target genes were used as follows: CHOP: forward 5′- ATGTGCGTGTGACCTCTGTT-3′ and reverse 5′-TATCTCATCCCCAGGAAACG, PERK: forward 5′-TAGATGAAACCAAGGAACCAGA-3′, and reverse 5′-ATCAGCACTTTAGATGGACGAA-3′). We amplified 2 μl cDNA for each sample in a 20 μl reaction system containing SYBR Green Master Mix and primers with the following cycling conditions: 2 min at 95°C, 40 cycles of 95°C for 10 s, then 60°C for 10 s. Expression levels were normalized with GAPDH in the same samples using the 2^−ΔΔCt^ method.

### Western Blot Analysis

RIPA lysis buffer (Beyotime) containing phenylmethylsulfonyl fluoride, protease inhibitor, and phosphatase inhibitor was used to crack renal tissues or cells. Total protein was isolated according to the standard methods (34). The protein was measured by Pierce BCA Protein Quantitation Kit (Pierce, Rockland, USA). Twenty five micrograms of protein from TCMK-1 cells or kidney cortex was separated on a 12–15% (w/v) polyacrylamide denaturing gel and electronically blotted onto 0.45 μm polyvinylidene difluoride (PVDF) membranes (Roche Diagnostics GmbH, Mannheim, Germany) on 14 volts for 16 h at 4°C or 100 volts for 1 h at room temperature. After 2-h blocking with 5% milk, caspase-3 or CHOP antibody (both at 1:200, Santa Cruz Biochemicals, Santa Cruz, USA), or PERK, JNK, HMGB-1, β-actin or α-Tubulin antibody (all at 1:1,000, Cell Signaling Technology, Beverly, USA) was incubated overnight at 4°C. The horseradish peroxidase-conjugated secondary antibody (Jackson ImmunoResearch Laboratories, West Grove, USA) was then incubated for 2 h at room temperature. Antibody binding was detected by enhanced chemiluminescent (ECL, Pierce) and a Molecular Imager, Chemi Doc, XRS+ System (Bio-Rad, Berkeley, USA) (36). Developed images were semi quantitatively analyzed by scanning volume density using Alpha View Software 3.3 (Cell Biosciences Inc., Santa Clara, USA). The expression of target protein was normalized against to the expression of β-actin or α-Tubulin, and presented as an ratio.

### Determination of Apoptosis by Flow Cytometry

Adherent TCMK-1 cells were harvested by tripsinizing and centrifuged at 1,000 rpm/min for 5 min, then washed twice with PBS. The pellet was resuspended in 100 μl of 1× binding buffer and incubated with 5 μl of FITC-conjugated annexin-V and 5 μl of propidium iodide (PI) for 15 min at room temperature in the dark. Another three tubes: annexin-V only, PI only and none dye was prepared as controls. Four hundred μl of 1× binding buffer was added to each sample tube, and the samples were analyzed by BD FACS Calibur flow cytometer (Becton Dickinson, San Jose, USA) using Cell Quest Research Software (BD Biosciences, Franklin Lakes, USA), a minimum of 10,000 cells were counted.

The results were shown as quadrant dot plots with survival cells (Annexin V-/PI-), early apoptotic cells (Annexin V+/PI-), later apoptotic cells or necrotic cells (Annexin V+/PI+). The number of each type of cells was expressed as the percentage of each type of cells against the total number of gated cells.

### Correlation Analysis

The correlation analysis was used to measure the correlation between two parameters. The range of R value between 0 and 1 or −1 and *P* < 0.05 implies positive or negative correlation (38). This method was used to analyze the correlation between CHOP and PERK, JNK, apoptosis, or cell viability in the *in vitro* model.

### Statistical Analysis

All data are representative of at least three independent experiments, while the data are presented as the mean ± standard error of the mean (SEM). The results among three or more groups were compared by one-way analysis of variance (ANOVA), while those in any two groups were compared using a two-tailed independent Student's *t*-test. All statistical analysis were performed using GraphPad Prism 6.0 (GraphPad Software Inc., San Diego, USA), with *P* < 0.05 considered statistically significant.

## Results

### ERS Induced by H_2_O_2_ in TCMK-1 Cells

**Figure 1:**
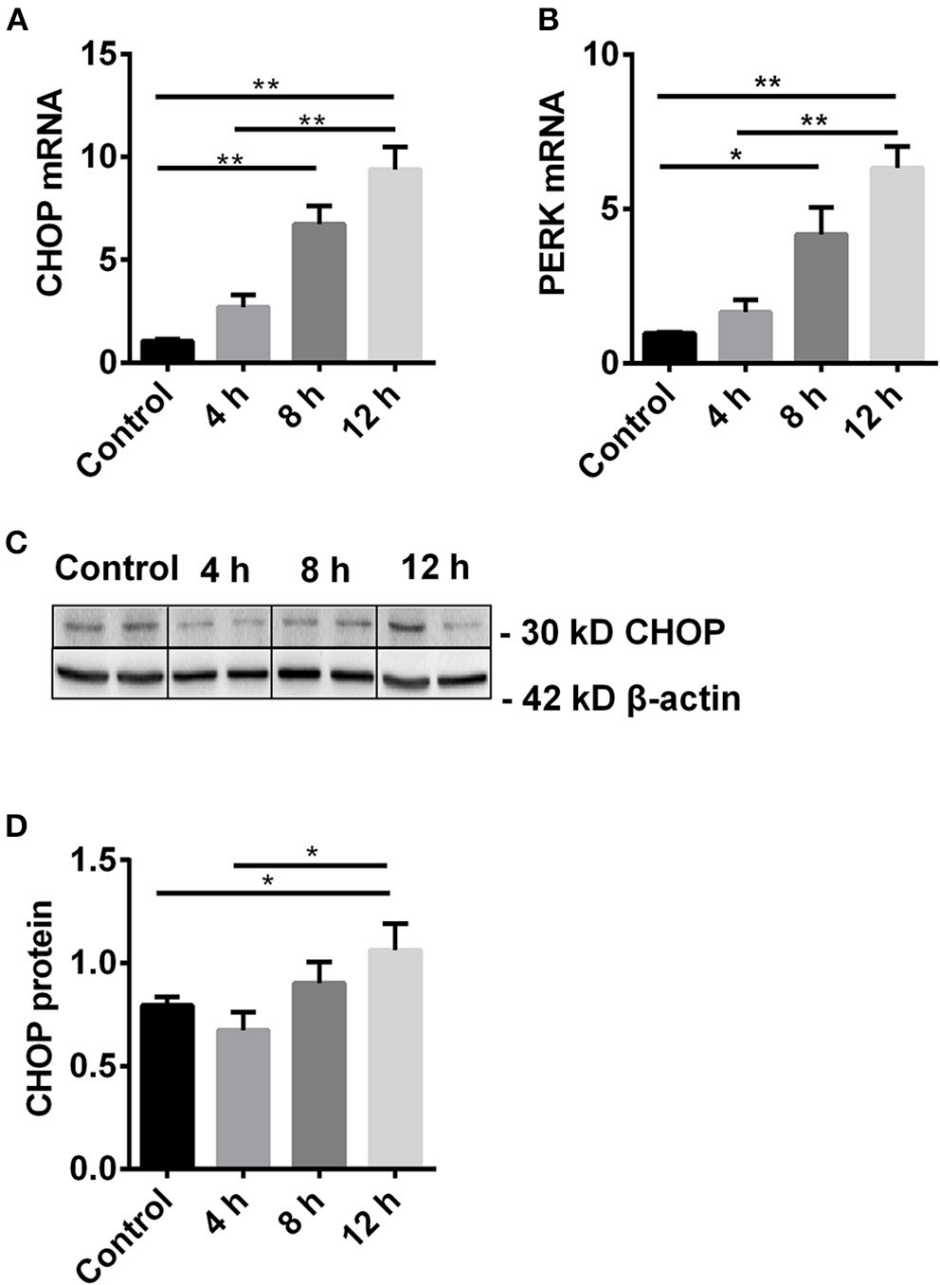
The dynamic change of CHOP and its regulator PERK. **(A,B)** The mRNA level of CHOP and PERK was gradually increased and reached the significance at 8 h and peaked at 12 h. **(C,D)** The expression of CHOP protein was significantly increased at 12 h. Data are expressed as mean ± SEM (*n* = 3). The volume density of western blots was corrected by against 42 kD β-actin as a loading control. *P < 0.05, **P < 0.01. h, hours.

To study the dynamic change of CHOP and its regulator PERK, the expression of CHOP mRNA and protein, and *PERK* mRNA was measured by qPCR and western blot in TCMK-1 cells treated with 500 μmol/L H_2_O_2_ for 4, 8, 12 h. The mRNA expression of CHOP and PERK was gradually increased with the time, and reached significance at 8 h compared with the control, and peaked at 12 h compared with the control or 4 h (all *P* < 0.01, Figures 1A,B). The expression of CHOP protein was also increased at 12 h (1.06 ± 0.13) compared with the control or 4 h, respectively (0.79 ± 0.04 or 0.67 ± 0.10, both *P* < 0.05, Figures 1C,D).

### CHOP siRNA Downregulated Its mRNA and Protein *in vitro*

**Figure 2:**
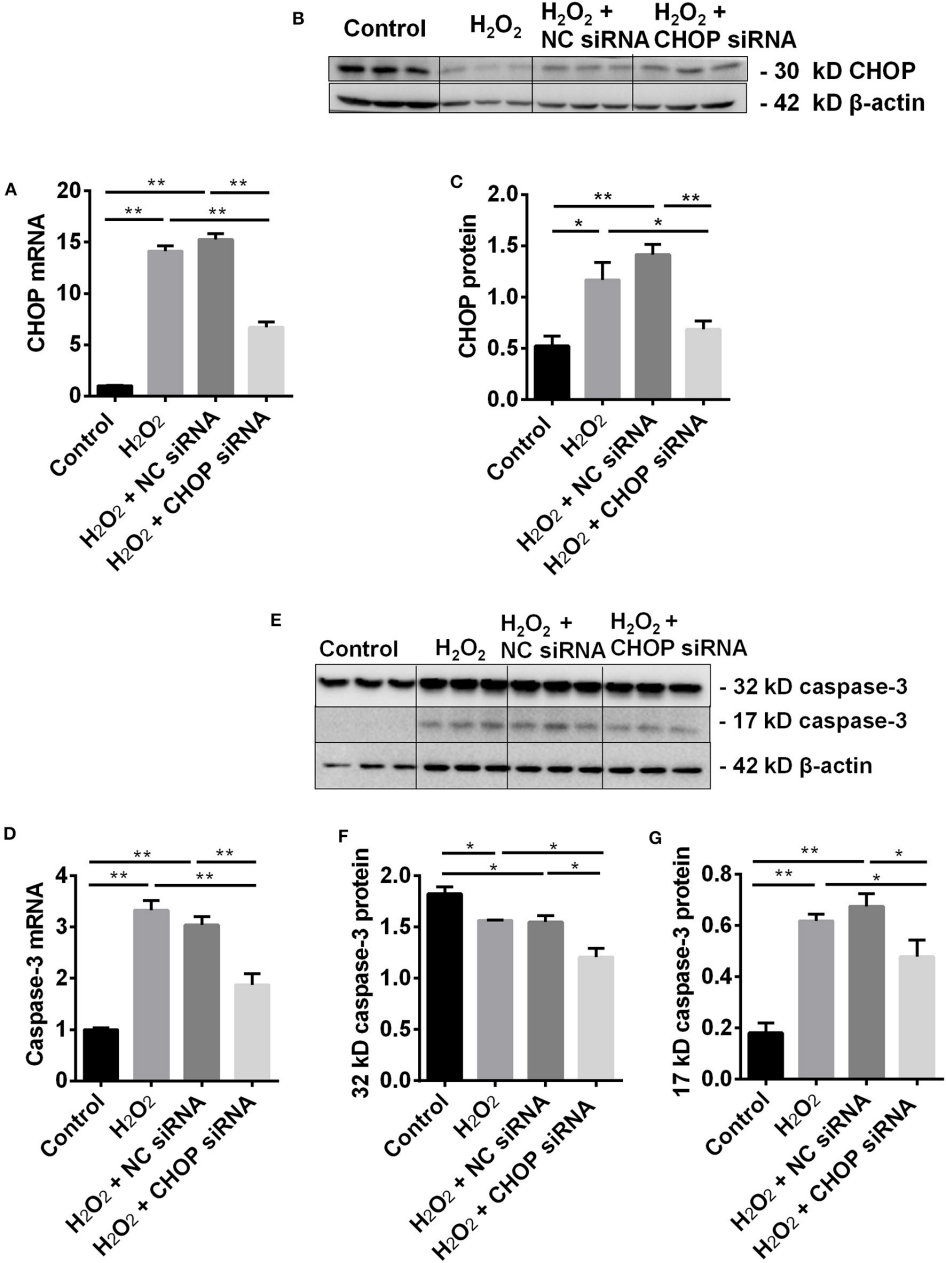
CHOP siRNA downregulated its mRNA, protein and caspase-3 in TCMK-1 cells. **(A)** The expression of *CHOP* mRNA in the TCMK-1 cells transfected with CHOP siRNA was reduced by 53% and 56% compared to the cells treated by the H_2_O_2_ and the NC siRNA. **(B,C,E,G)** The expression of CHOP protein as well as *caspase-3* mRNA **(D)** and 17 kD caspase-3 protein was increased at the H_2_O_2_ and the NC siRNA group but reversed by CHOP siRNA. **(E,F)** The expression of 32 kD caspase 3 protein was decreased by H_2_O_2_ or NC siRNA and further decreased by CHOP siRNA. Data are expressed as mean ± SEM (*n* = 3). The volume density of western blots was corrected by against 42 kD β-actin as a loading control. *P < 0.05, **P < 0.01.

In the TCMK-1 cells transfected with CHOP siRNA, the expression of *CHOP* mRNA was reduced by 53% and 56% compared to the cells treated by H_2_O_2_ or the negative control (NC) siRNA, respectively (both *P* < 0.01, Figure 2A). In addition, CHOP siRNA also reduced the expression of CHOP protein (0.69 ± 0.08 vs. 1.17 ± 0.17 or 1.42 ± 0.10, *P* < 0.05 or 0.01, Figures 2B,C) in the same comparisons.

### CHOP siRNA Decreased Caspase-3 and HMGB-1 Expression in TCMK-1 Cells

The effect of CHOP siRNA on caspase-3 expression was further investigated. The level of *caspase-3* mRNA (Figure 2D) and 17 kD protein (Figures 2E,G), contrary to 32 kD protein (Figures 2E,F), was increased in the H_2_O_2_ or NC siRNA group. However, they all were significantly decreased by CHOP siRNA (mRNA: 1.88 ± 0.21 vs. 3.33 ± 0.19 or 3.04 ± 0.17, 17 kD: 1.21 ± 0.08 vs. 1.57 ± 0.01 or 1.55 ± 0.07, 32 kD: 0.34 ± 0.04 vs. 0.62 ± 0.03 or 0.60 ± 0.07, *P* < 0.05 or 0.01).

**Figure 3:**
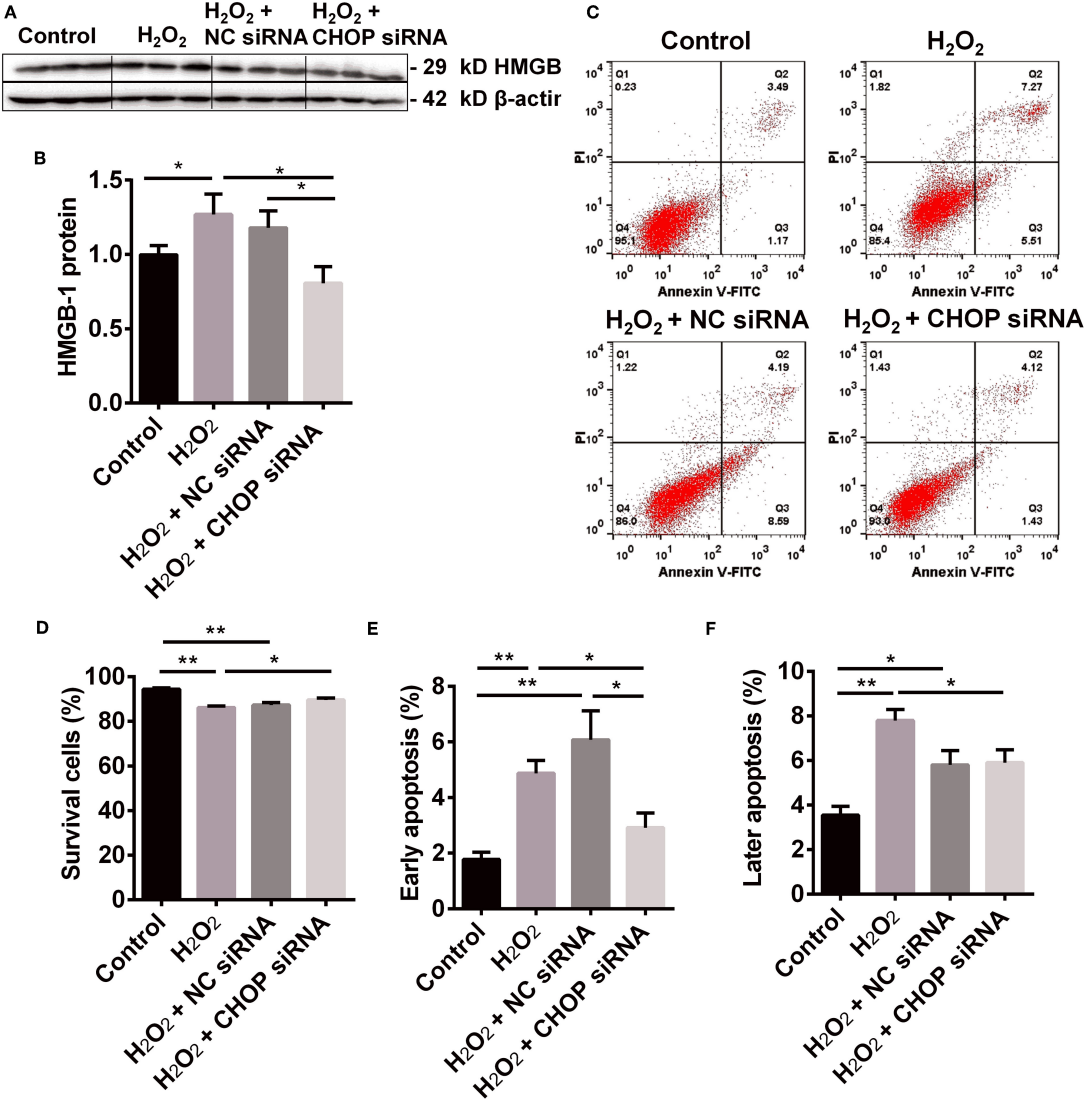
The renoprotection of CHBP associated with ERS improvement, apoptosis and inflammation. **(A,B)** HMGB-1 protein was significantly up-regulated by H_2_O_2_, but was down-regulated by CHOP siRNA compared to the H_2_O_2_ and NC siRNA groups. **(C,D)** The percentage of living cells was decreased by H_2_O_2_ and H_2_O_2_ + NC siRNA but reversed by H_2_O_2_ + CHOP siRNA. **(E,F)** The both early and later apoptotic cells were increased by H_2_O_2_ and H_2_O_2_ + NC siRNA, but only the early apoptotic cells were reduced by H_2_O_2_ + CHOP siRNA. Data are expressed as mean ± SEM (*n* = 3). The volume density of western blots was corrected by against 42 kD β-actin as a loading control. *P < 0.05, **P < 0.01.

The level of HMGB-1 protein was significantly up-regulated in the H_2_O_2_ group (*P* < 0.05, Figures 3A,B), but was down-regulated by CHOP siRNA compared to the H_2_O_2_ and NC siRNA group (0.81 ± 0.11 vs. 1.27 ± 0.34 and 1.18 ± 0.11, *P* < 0.05).

### CHOP siRNA Decreased Apoptosis in TCMK-1 Cells

The cells stimulated by H_2_O_2_, NC siRNA or CHOP siRNA post H_2_O_2_ stimulation were presented (Figure 3C). The percentage of survival cells was decreased by H_2_O_2_ and NC siRNA compared with the control group, but reversed by CHOP siRNA (81.55 ± 1.74 vs. 75.66 ± 1.54, *P* < 0.05, Figure 3D). The early apoptotic cells and later apoptotic cells were increased, respectively, by H_2_O_2_ and NC siRNA treatment compared with the control group (Figures 3E,F). Only the early apoptotic cells were reduced by CHOP siRNA compared to the H_2_O_2_ and NC siRNA group (7.07 ± 1.28 vs. 14.58 ± 1.71 and 16.84 ± 2.74, *P* < 0.01, and 0.05).

### CHOP Correlated With PERK, Caspase-3, HMGB-1, and Apoptosis Positively, but Cell Viability Negatively

**Figure 4:**
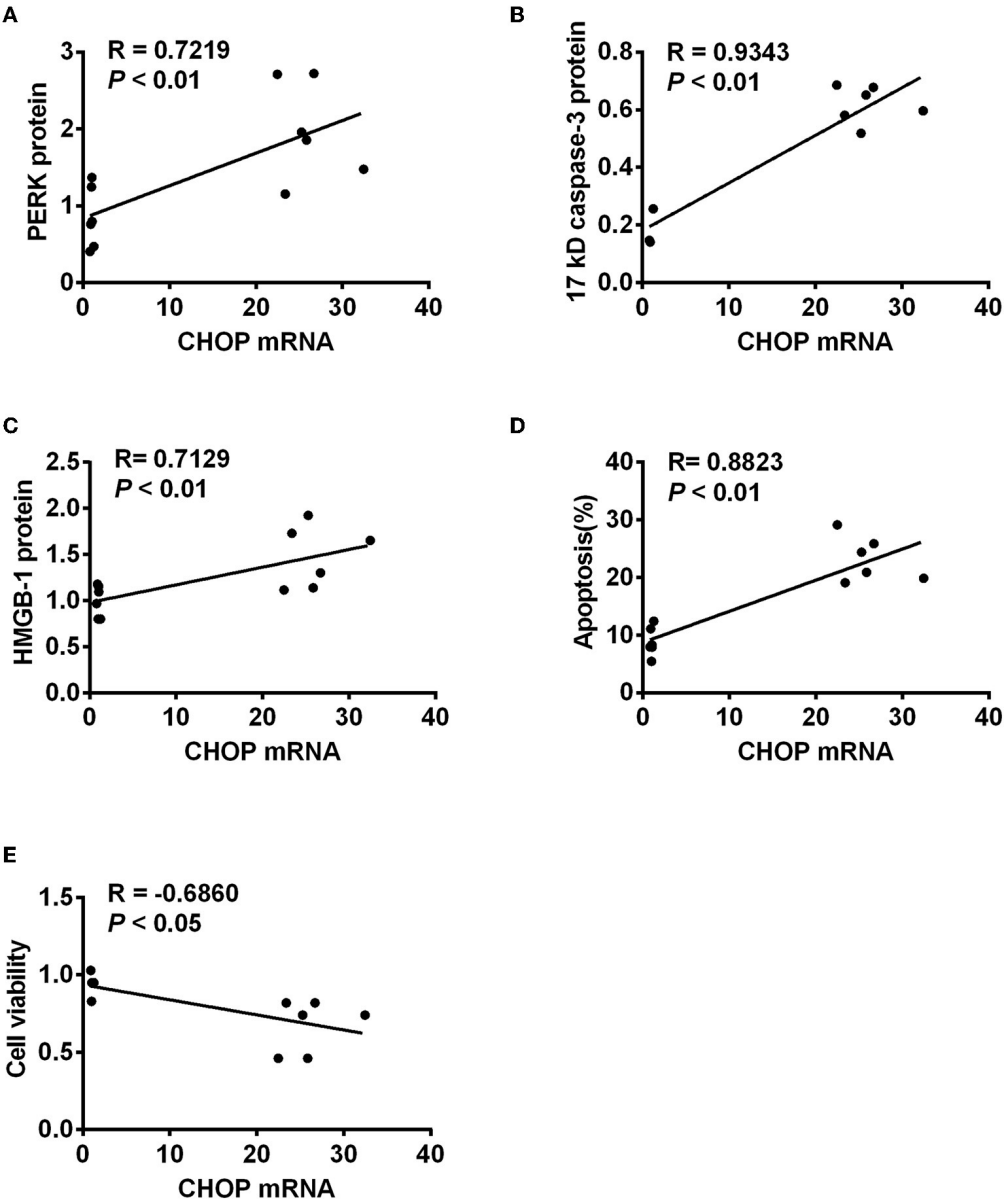
The correlation between *CHOP* mRNA and other detected parameters. *CHOP* mRNA was positively correlated with PERK protein **(A)**, active caspase-3 **(B)**, HMGB-1 **(C)**, and apoptosis **(D)** (*R* = 0.7219, 0.9343, 0.7129, and 0.8823, all *P* < 0.01) but negatively with cell viability (**E**, *R* = −0.5248, *P* < 0.05) in the *in vitro* model.

To further investigate the role of CHOP in IR-related injuries, the correlation between *CHOP* mRNA and PERK, 17 kD active caspase-3, HMGB-1, apoptosis, and cell viability was analyzed, respectively. *CHOP* mRNA was correlated positively with PERK protein, active caspase-3, HMGB-1 and apoptosis (*R* = 0.7219, 0.9343, 0.7129, and 0.8823, all *P* < 0.01, Figure 4), but negatively with cell viability (*R* = −0.5248, *P* < 0.05) in the *in vitro* model.

### CHBP Increased TCMK-1 Cell Viability Post H_2_O_2_ Stimulation

**Figure 5:**
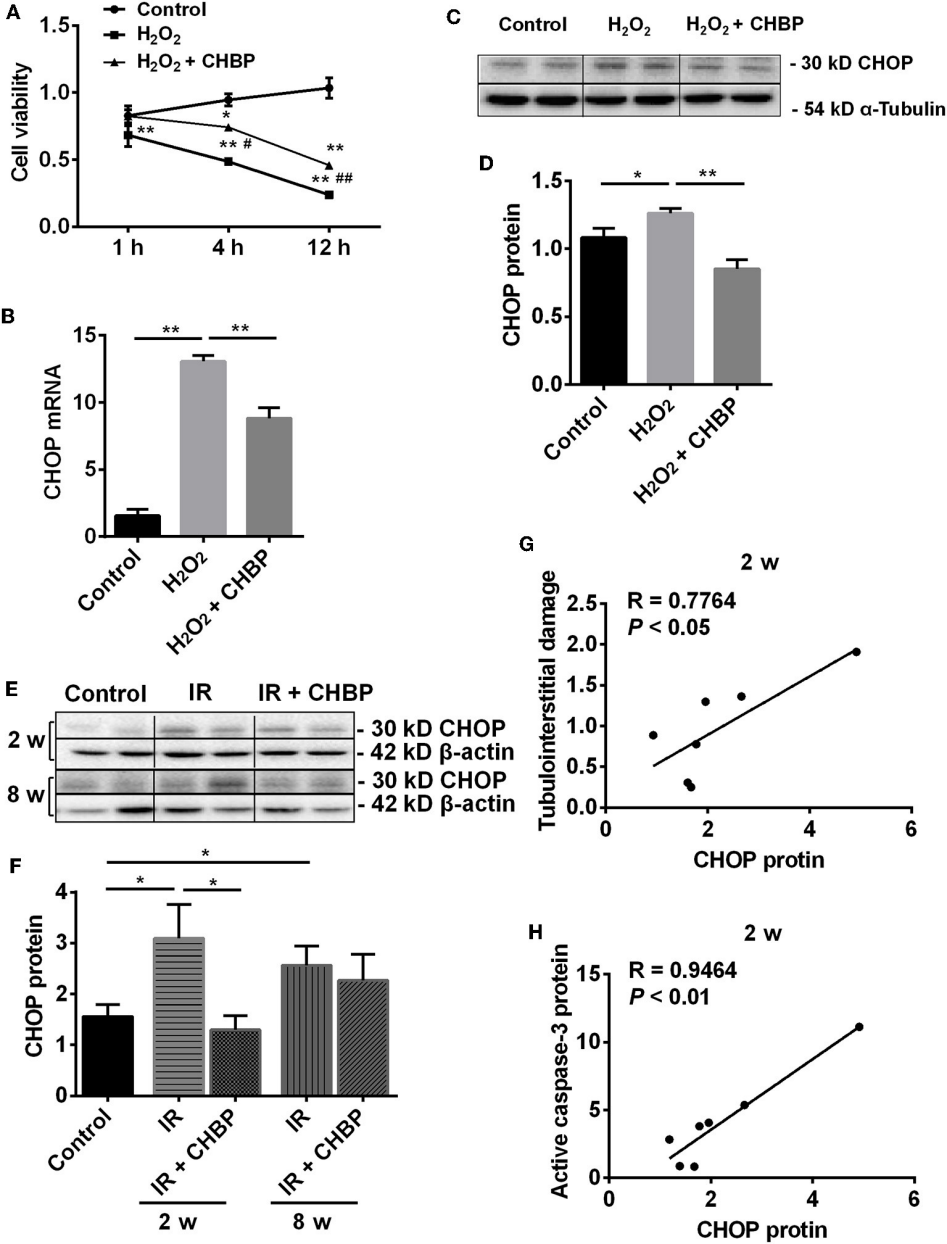
The dynamic change of cell viability and the expression of CHOP mRNA and protein in TCMK-1 cells and kidneys. **(A)** The cell viability was gradually decreased with the time of H_2_O_2_ treatment, reached the significance from 1 to 12 h, but was significantly improved in the H_2_O_2_ + CHBP group after 4 h. **(B–D)** The mRNA and protein of CHOP were significantly increased in the TCMK-1 cells stimulated by H_2_O_2_, but reversed by CHBP. **(E,F)** CHOP protein was also significantly increased in the IR group at 2 and 8 weeks, but was decreased by CHBP only at 2 weeks. Data are expressed as mean ± SEM (*n* = 3–6). The volume density of western blots was corrected by against 42 kD β-actin or 54 kD α-Tubulin as a loading control. **(G,H)** CHOP protein was positively correlated with the level of TID and active caspase-3 protein at 2 weeks. *, #*P* < 0.05, **, ##*P* < 0.01 vs. Control, 4 h or H_2_O_2_ + CHBP.

In order to observe the effect of CHBP, renoprotective peptide derived from erythropoietin, on cell viability, the CCK-8 assay was used to detect the value of optical density (OD) at 1, 4, 12 h after H_2_O_2_ stimulation (Figure 5A). The cell viability gradually decreased with the time of H_2_O_2_ stimulation with or without CHBP and reached the significance from 1 to 12 h compared with the control (*P* < 0.01 or 0.05).

More importantly, the cell viability was significantly increased in the H_2_O_2_ + CHBP group at 4 and 12 h (0.74 ± 0.08 and 0.46 ± 0.06) compared with the H_2_O_2_ group (0.49 ± 0.06 and 0.24 ± 0.04, *P* < 0.05 and 0.01).

### CHBP Reduced ERS Induced by H_2_O_2_ and IR Injury

To further dissect the mechanism of CHBP renoprotection in association with ERS, the mRNA and protein of CHOP were measured in the TCMK-1 cells stimulated by H_2_O_2_ for 12 h (Figures 5B–D). It has been revealed that the mRNA and protein of CHOP were increased (*P* < 0.01 or 0.05), whereas the increased CHOP mRNA and protein were reversed by CHBP treatment (mRNA: 8.82 ± 0.79 vs. 13.06 ± 0.44, protein: 0.85 ± 0.06 vs. 1.26 ± 0.04, *P* < 0.01).

In addition, the expression of CHOP protein was significantly increased in the IR kidneys at 2 and 8 weeks (both *P* < 0.05), which was decreased by CHBP only at 2 weeks (1.30 ± 0.28 vs. 3.10 ± 0.66, *P* < 0.05, Figures 5E,F). The CHOP protein was also correlated positively with the level of tubulointerstitial damage (TID) and active caspase-3 protein at 2 weeks (*R* = 0.7764 and 0.9464, *P* < 0.05 or 0.01, Figures 5G,H). Besides that, a trend of positive correlation between CHOP protein and apoptosis or interstitial fibrosis was showed at 2 weeks, respectively, even though there was no statistical significance (*R* = 0.5612, *P* = 0.1478; *R* = 0.4467, *P* = 0.2671). However, the original data of TID, active caspase-3 protein, apoptosis and interstitial fibrosis have not been shown here.

**Figure 6:**
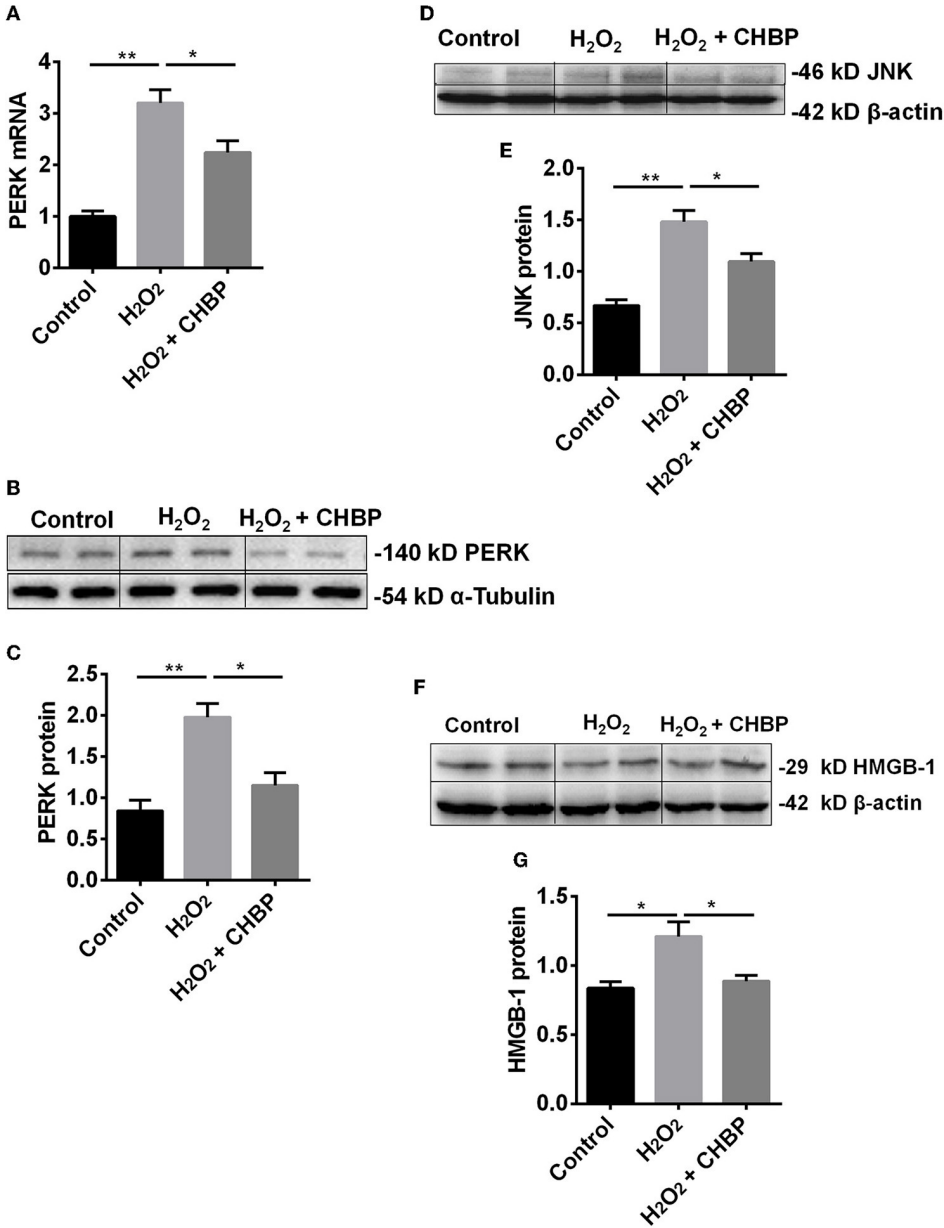
The expression of PERK, JNK and HMGB-1 protein and/or mRNA. The mRNA **(A)** and protein **(B,C)** of PERK, as well as JNK protein **(D,E)** and HMGB-1 protein **(F,G)**, were significantly increased by H_2_O_2_ in the TCMK-1 cells. However, these effects were reversed by CHBP. Data are expressed as mean ± SEM (*n* = 3). The volume density of western blots was corrected by against 42 kD β-actin or 54 kD α-Tubulin as a loading control. *P < 0.05, **P < 0.01.

Moreover, the level of *PERK* mRNA and protein (Figures 6A–C), JNK protein (Figures 6D,E) and HMGB-1 protein (Figures 6F,G) was significantly up-regulated by H_2_O_2_ (*P* < 0.05 or 0.01), which was down-regulated by CHBP (*PERK* mRNA: 2.24 ± 0.22 vs. 3.20 ± 0.26, PERK protein: 1.15 ± 0.15 vs. 1.98 ± 0.17, JNK protein: 1.10 ± 0.08 vs. 1.48 ± 0.11, HMGB-1 protein: 0.89 ± 0.04 vs. 1.21 ± 0.11, all *P* < 0.05).

### CHBP Inhibited Cell Apoptosis *in vitro*

**Figure 7:**
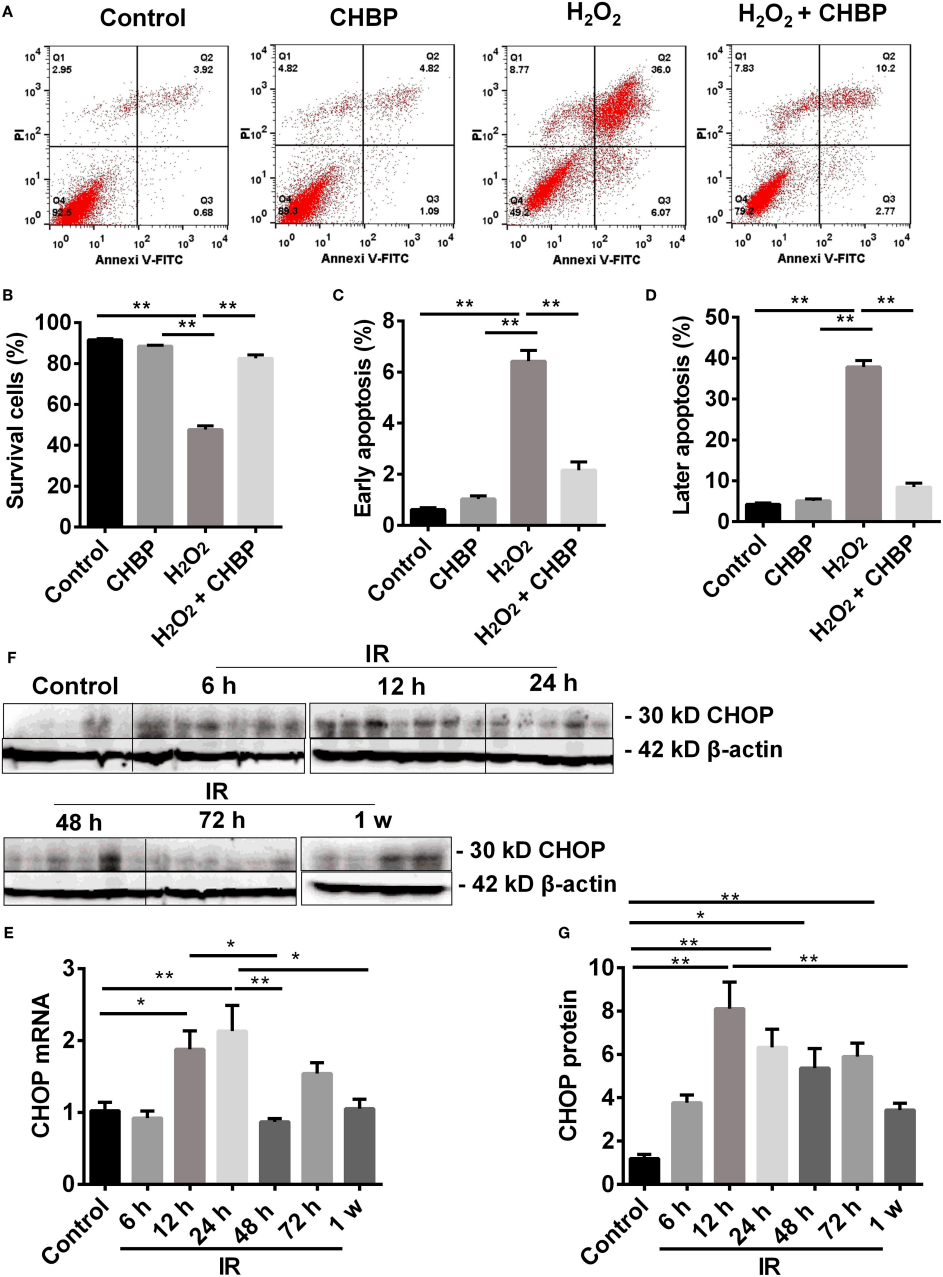
Cell apoptosis analyzed by flow cytometry and CHOP protein detected by western blot. (**A,B**: Annexin V-FITC-/PI-) The percentage of living cells was decreased by H_2_O_2_, but reversed by CHBP. (**A,C**: Annexin V +/PI-; **A,D**: Annexin V +/PI+) The early apoptotic cells, as well as later apoptotic cells were increased, respectively, by H_2_O_2_, but reduced by CHBP. **(E–G)** The dynamic expression of CHOP at mRNA and protein level. **(E,F)** CHOP protein was significantly raised and picked at 12 h. **(G)** CHOP mRNA was also significantly increased at 12 h and peaked at 24 h after IR injury. Both CHOP protein and mRNA were decreased at 1 week. Data are expressed as mean ± SEM (*n* = 3). The volume density of western blots was corrected by against 42 kD β-actin as a loading control. *P < 0.05, **P < 0.01.

Apoptotic cells were detected by flow cytometry using Annexin V/PI staining. The population of cells in the control, H_2_O_2_, CHBP, or CHBP post H_2_O_2_ stimulation group was presented (Figure 7A). The percentage of survival cells (Annexin V-/PI-) was decreased by H_2_O_2_ treatment compared with the control groups with or without CHBP treatment, but effectively reversed by CHBP (88.15 ± 0.57 vs. 43.23 ± 2.88, *P* < 0.01, Figure 7B). Both the early apoptotic cells (Annexin V+/PI-) and later apoptotic cells (Annexin V+/PI+) were increased, respectively, by H_2_O_2_ treatment compared with the control group and the CHBP group (Figures 7C,D), but reduced by CHBP almost to the baseline level (1.85 ± 0.22 vs. 6.67 ± 0.13, 7.88 ± 0.61 vs. 45.55, both *P* < 0.01).

### Dynamic Change of CHOP at Different Time Post IR Injury

The expression of CHOP mRNA was significantly increased from 12 h after IR injury, peaked at 24 h, and then significantly decreased at 48 h compared with both 12 and 24 h, and also at 1 week compared with 24 h. CHOP protein was also significantly raised at 12, 24, 48, and 72 h compared with the control with a peak at 12 h, and decreased at 1 week compared with 12 h (*P* < 0.05 or 0.01, Figures 7E–G).

## Discussion

**Figure 8:**
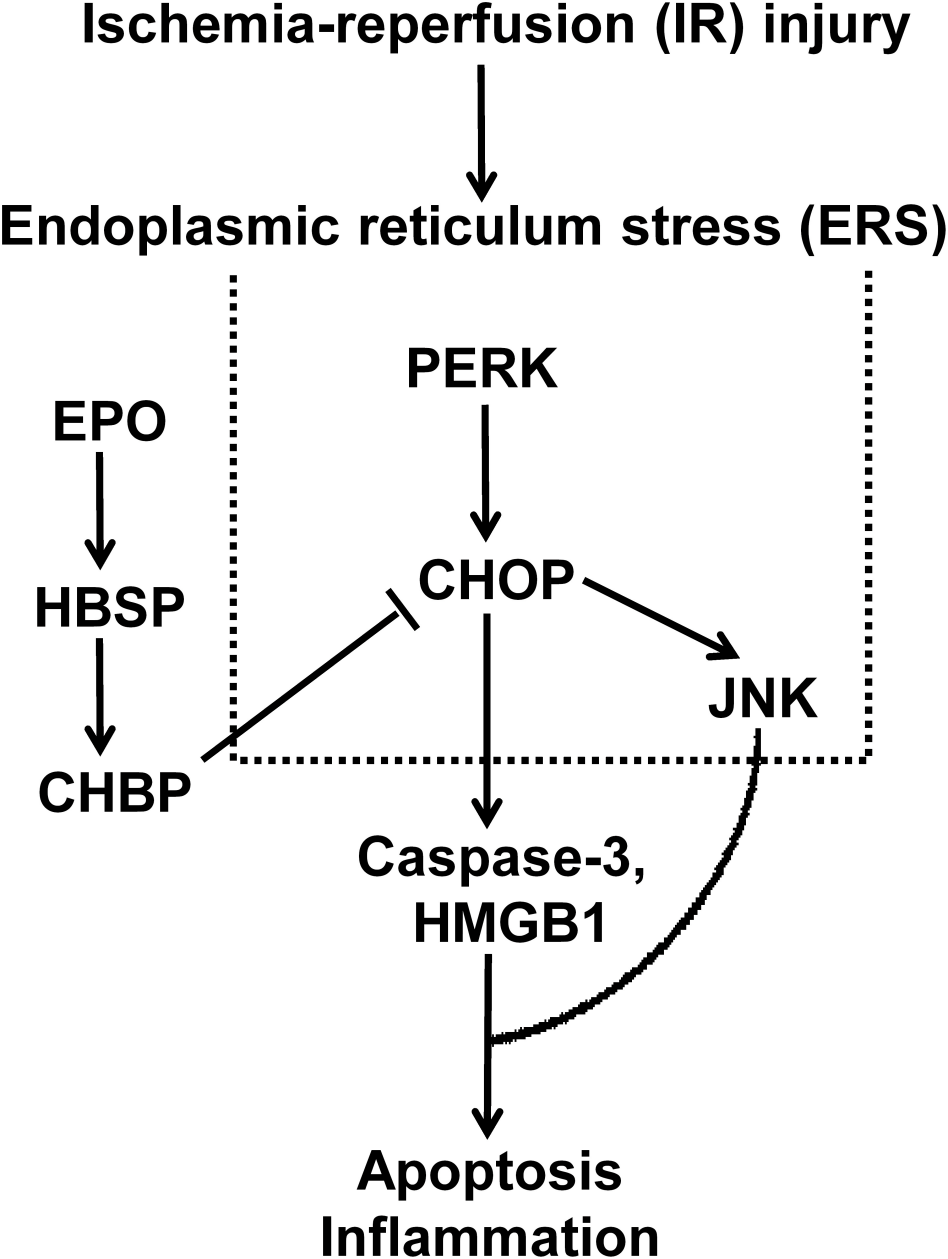
The schematic illustration shows that the key role of ERS in IR-related injury, and CHBP-incuced renoprotection was associated with CHOP/PERK/JNK, HMGB-1/caspase-3, and apoptosis.

The role and its underlying mechanism of ERS in IR-related injury and CHBP-induced renoprotection have been well-investigated in this study using *in vitro* and *in vivo* models. The expression of CHOP, a key marker of ERS, was increased in IR-related and IR injury. In addition, the intervening CHOP using siRNA reduced CHOP expression at both mRNA and protein level, subsequent decreased caspase-3 and HMGB-1 protein, and early apoptosis. Furthermore, CHBP treatment significantly improved ERS, in terms of decreased CHOP expression in TCMK-1 cells and kidneys subjected to IR-related and IR injury, as well as its regulators PERK and JNK, apoptotic and inflammatory mediator HMGB-1, and early and later apoptosis *in vitro*. Therefore, the key role of ERS in IR-related injury and CHBP induced renoprotection was proved in both mouse TCMK-1 cells and kidneys, in which its mechanistic signaling pathway was associated with CHOP/PERK/JNK, HMGB-1/caspase-3, and apoptosis (Figure 8).

The dynamic change of *CHOP* and *PERK* mRNA was evaluated in TCMK-1 at different time points after H_2_O_2_ stimulation. The consequential increase of these key parameters was observed, which reflected the gradual occurrence of ERS. The expression of CHOP protein was also increased significantly with time from 8 to 12 h compared with the control and 4 h. The change of CHOP at mRNA level represents transcriptional change, while that of CHOP at protein level represents translational change. The divergence of both at 4 h reflected differential regulations between transcription and translation. In addition, the temporary decline of CHOP protein at 4 h may be due to consumption involved in apoptosis, a self-protective regulation of cells, which subsequently stimulated the increase of CHOP at mRNA. It has been confirmed that 500 μmol/L H_2_O_2_ for stimulation over a period of 12 h were the most suitable experimental condition for the following investigation.

CHOP is a key player in ERS-mediated apoptotic pathway (20, 39). In response to severe ERS, CHOP activates the expression of Bim, leading to caspase-3 cleavage and apoptosis (40). Caspase-3 associated with apoptosis and inflammation involved in renal IR-related and IR injury. The precursor of caspase-3 could be cleaved into 17 and 12 kD subunits, both of which contribute to caspase-3 activity (34, 37, 41, 42). The changes of CHOP activate the ER-associated caspase cascades, including caspase-12 and caspases-3 (37). Therefore, we further investigate the effect of CHOP intervention by its siRNA on apoptotic and inflammatory mediator caspase-3 and HMGB-1, and apoptosis in renal IR-related injury. CHOP siRNA decreased the expression of CHOP mRNA and protein, as well as *caspase-3* mRNA, 32 and 17 kD caspase-3, and HMGB-1 protein. Moreover, the number of early apoptotic cells was also reduced by CHOP siRNA compared to the H_2_O_2_ and NC siRNA groups, and the cell survival was improved. These data were consistent to the CHOP^−/−^ knockout model *in vivo* (21, 43, 44). Furthermore, the correlation analysis revealed that *CHOP* mRNA was positively correlated with PERK, 17 kD active caspase-3 and HMGB-1, and apoptosis, but negatively related to cell viability. These results were in accordance with some other studies (40, 45, 46). Therefore, our data convincingly proved that CHOP was involved in the process of apoptosis, especially in its early stage, in renal IR-related injury.

The decreased cell viability upon H_2_O_2_ stimulation, in addition, was significantly reversed by CHBP treatment time-dependently, which confirmed the cellular protection of CHBP. More interestingly, CHBP also reduced CHOP mRNA and protein expression in TCMK-1 cells, while increased CHOP protein expression in the IR kidneys at prolonged time points was also decreased by CHBP treatment at 2 weeks, even though not at 8 weeks. Therefore, our data demonstrated that CHOP involved in renal IR-related and IR injuries, and CHBP-induced renoprotection might be through regulating CHOP or improving ERS. However, when UPR response cannot restore the homeostasis of endoplasmic reticulum upon IR injury, PERK pathway could trigger off downstream apoptosis via the activation of CHOP, caspase-12 and Bcl-2 family (44, 47, 48). Additional correlation analysis revealed that CHOP protein was positively correlated with the level of TID and active caspase-3 protein at 2 weeks. A trend of positive correlation between CHOP protein and interstitial fibrosis, apoptosis was also revealed at 2 weeks. But because the sample size is insufficient, it was not reached statistical significance. Therefore, CHBP might be renoprotective against IR-induced apoptosis and tubular damage mediated by CHOP, which could be more effective at 2 weeks than 8 weeks, as the level of CHOP could be alleviated with the time post IR injury naturally. These results also indicate that the efficacy of CHBP renoprotection might depend on the level of CHOP in certain degree, the higher of ERS, the better of CHBP renoprotection. In order to study the underlying relationship between CHBP and ERS, the changes in two main up and down-stream regulators of CHOP, PERK, and JNK, were further explored. PERK mRNA and protein, and JNK protein, were all increased significantly in the TCMK-1 cells stimulated by H_2_O_2_, but was reversed by CHBP treatment. These results imply that the renoprotection of CHBP was attributed to the improvement of CHOP associated ERS, especially at the early stage of renal IR injury. Furthermore, both early and later apoptotic cells increased by H_2_O_2_ stimulation all reversed by CHBP treatment that was more potent than CHOP siRNA. However, there was no impact of CHPB on apoptosis at baseline level. The change of HMGB-1 protein (49), a mediator of apoptosis and inflammation, has a similar trend with that of CHOP, PERK, and JNK *in vitro*. These results revealed that the protective effects of CHBP were related to not only ERS improvement, but also apoptotic and inflammatory mediator, and apoptosis. To confirm whether ERS plays key roles at the early stage of IR injury, we established a time course model with reperfusion at 6, 12, 24, 48, 72 h, and 1 week. The dynamic change of CHOP showed that the expression of CHOP mRNA and protein was significantly increased as early as 12 h post IR injury peaked at 24 and 12 h respectively, and then both decreased at 1 week. Therefore, the intervention at early time points is worth to be further investigated.

In conclusion, ERS and CHBP play differential roles in IR-related injury in TCMK-1 cells and kidneys. The mechanistic signaling pathway is associated with CHOP/PERK/JNK, HMGB-1/caspase-3, and apoptosis. This study, therefore, indicates that CHOP might be a potential biomarker for diagnosis and treatment of IR-induced acute kidney injury, and CHBP might also have therapeutic application clinically.

## Data Availability Statement

The datasets generated for this study are available on request to the corresponding author.

## Ethics Statement

The animal study was reviewed and approved by Ethics committee of affiliated hospital of Nantong University.

## Author Contributions

BY, YF, and TZ conceived and designed the study. YZ, QW, AL, YW, FL, and HW acquired, analyzed, and interpreted the data. YZ and BY wrote the paper. BY final approval of submitted version. All authors participated in the design, interpretation of the studies and analysis of the data and review of the manuscript.

### Conflict of Interest

The authors declare that the research was conducted in the absence of any commercial or financial relationships that could be construed as a potential conflict of interest.
